# Effectiveness of Essence of Chicken in Improving Cognitive Function in Young People Under Work-Related Stress: A Randomized Double-Blind Trial

**DOI:** 10.1097/MD.0000000000003640

**Published:** 2016-05-13

**Authors:** Lung Chan, Hsuan-Min Wang, Kuan-Yu Chen, Ying-Chin Lin, Pei-Jung Wu, Wan-Lin Hsieh, Ying-Ru Chen, Cheung-Pin Liu, Han-Yin Tsai, Yun-Ru Chen, Hsiu-Hui Chang, Yi-Chen Hsieh, Chaur-Jong Hu

**Affiliations:** From the Department of Neurology (LC, K-YC, P-JW, C-JH), Shuang-Ho Hospital, School of Medicine, College of Medicine, Taipei Medical University, New Taipei City; Department of Psychiatry (H-MW), Chang Gung Memorial Hospital, Chiayi; Department of Family Medicine (Y-CL, Y-RC, C-PL), Shuang-Ho Hospital, School of Medicine, College of Medicine, Taipei Medical University; Department of Psychiatry (W-LH); Sleep Center (H-YT); Health Management Center (Y-RC), Shuang-Ho Hospital, School of Medicine, Taipei Medical University, New Taipei City; Department of International Trade (H-HC), Chungyu Institute of Technology, Keelung; Graduate Institute for Neural Regenerative Medicine (Y-CH), College of Medical Science and Technology, Taipei Medical University, Taipei, Taiwan.

## Abstract

Work-related stress (WS) can result in considerable and extensive changes in physiological and psychological performance. WS beyond the optimal levels induces anxiety, confusion, exhaustion, and burnout. Chronic WS affects neurocognitive performance, particularly attention and visuospatial memory. Essence of chicken (EC) has been reported to improve neurocognitive function after mental stress.

To investigate the beneficial effects of EC in improving neurocognitive performance under WS, we conducted a randomized, double blind trial. Total 102 young workers in New Taipei City with high WS, evaluated using the Individual Subjective Perception Job Stress Scale scores (>36 for job leaders and 33 for nonleaders) were recruited. Fifty-one participants received 70 mL of EC and 51 received a placebo daily for 2 weeks. Blood tests and neurocognitive assessment were performed before treatment, at the end of treatment, and 2 weeks after treatment.

EC improved the performance of participants with high depression scores in the form-color associative memory test, used for assessing short-term memory. Although creatinine and glutamic-pyruvic transaminase (GPT) levels increased in week 2, but the levels returned to the baseline in week 4. Blood urea nitrogen (BUN) levels decreased in week 4.

EC significantly improved short-term memory in participants with high WS and concomitant depressive mood, although it slightly increased GPT and creatinine levels and reduced BUN levels. The long-term treatment effects of EC warrant further investigation.

## INTRODUCTION

The perception of a stimulus as overwhelming causes stress, which in turn elicits a measurable response resulting in a transformed state.^1^ Stress is usually caused by pressure or demand that affects a person's coping ability. Work-related stress (WS) usually occurs because of imbalance between the demands of a job and the resources and capabilities of the worker to meet these demands.^2^ WS can result in significant and extensive changes in physiological and psychological performance. Although stress can improve job efficiency, WS beyond the optimal levels induces anxiety, confusion, exhaustion, and burnout.^3–6^ Chronic WS affects neurocognitive performance, particularly attention and visuospatial memory, in which the frontal–medial temporal cortex network is involved. A previous study proposed decline in neurocognitive functions to be associated with low quality of life and a reduced adrenocorticotropic hormone (ACTH) response to corticotrophin-releasing hormone.^7^ In addition, chronic WS reduced brain volumes of the anterior cingulate cortex, dorsolateral prefrontal cortex, caudate, and putamen.^8^ Few reports have documented that stress might alter the immunity status. Levels of monocyte-produced antiinflammatory cytokine IL-10 increased in burnout syndrome, a severe consequence of WS.^9,10^ In a study on nurses working in an emergency department, the secretion rates of salivary IgA and lysozyme significantly decreased with an increase in WS levels.^11^

Essence of chicken (EC) is a liquid nutritional supplement prepared by cooking whole chickens for several hours at a high temperature. In traditional Chinese medicine, EC is used for strengthening the musculoskeletal system, spleen, and stomach and for enhancing vigor. EC contains minerals, vitamins, trace elements, indispensable amino acids, and nutrients such as carnosine and anserine.^12,13^ EC has been extensively studied and is known to affect multiple organ systems (immune,^14^ cardiovascular, and renal systems^15^) and physiological processes (erythropoiesis^16^ and circadian rhythm, specifically jet lag^17^) and reverses chemotherapy-induced bone marrow suppression.^18^ In previous reports, EC exerted antistress and antifatigue effects by regulating the cortisol levels and activating the brain histaminergic system.^19–21^ In stressed animals, EC alleviated stress-induced fatigue by preventing stress-mediated dysfunction caused by elevated insulin levels and increasing lipase activity and glycogen synthesis.^22^ Many clinical studies have showed the beneficial effects of EC on maintaining and enhancing neurocognitive performance, particularly attention and short-term memory under stressful conditions.^20,23^ The antistress effects of EC were attributed to enhancement of cortisol levels.^20^ On the basis of previous research, we hypothesize that EC can serve as a neurocognitive enhancer and antistress food, promote resilience and resistance to stress, and maintain neurocognitive functions. However, well-designed clinical trials, particularly randomized, double blind trials, for testing the effects of EC are lacking. Therefore, the aim of the current study was to investigate the beneficial effects of EC in improving neurocognitive performance under WS in a randomized, double blind trial.

## METHODS

### Study Design

**FIGURE 1:**
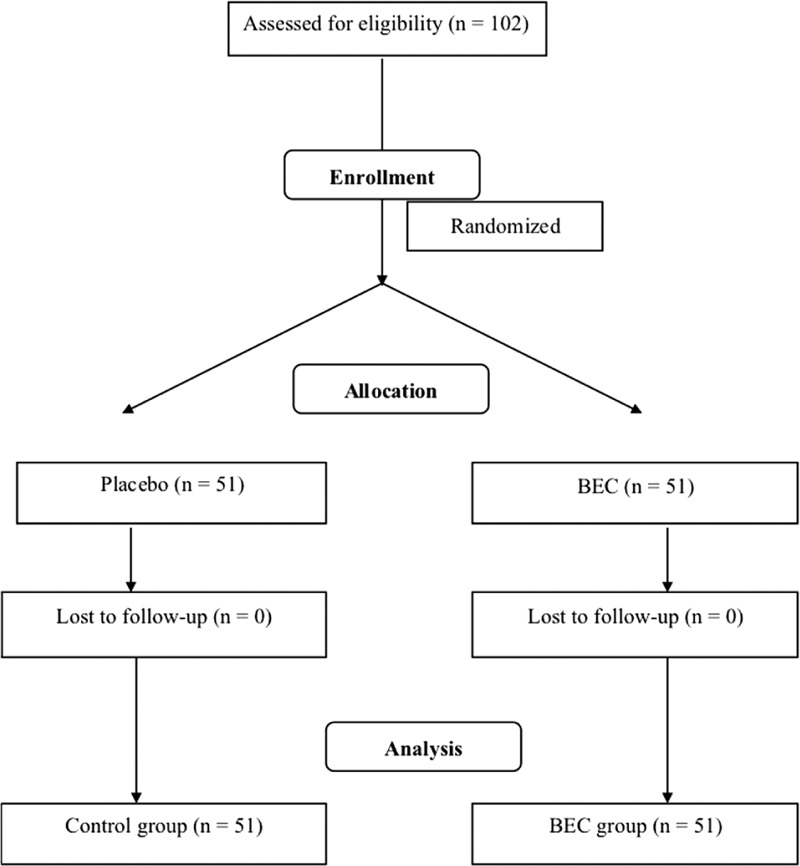
Summary of the randomized, double blind trial.

This study had a randomized, double blind trial design and was approved by the Joint Institutional Review Board of Taipei Medical University. The study was registered with the registration number NCT02166931. The participants and investigators were both blinded to the participant groups. Participants who met the inclusion criteria were randomly administered BRAND essence of chicken (BEC) or a placebo daily (70 mL) for 2 weeks after enrollment (Figure 1). Neurocognitive assessment and blood tests were performed at week 0 (before treatment), week 2 (at the end of treatment), and week 4 (2 weeks after treatment).

### Participants and WS Evaluation

All potential participants were evaluated using Individual Subjective Perception Job Stress Scale (ISPJSS) scores before randomization. The ISPJSS is a 20-item self-report questionnaire adapted from the Occupational Stress Scale created by the Institute of Occupational Safety and Health of Taiwan. The Cronbach alpha of this questionnaire is 0.96, and the coefficient of internal consistency is 0.87. The participants answered all items on a 4-point fully labeled Likert scale (0 = not much; 3 = very much), and their responses were summed to obtain a total score.^24^ The inclusion criteria were: an age of 20 to 65 years with at least 9 years of education and provision of written informed consent, no chronic systemic diseases treated using medication, a score >36 for job leaders and 33 for nonleaders on the ISPJSS,^24^ and no current use of medication that affects the mental state or neurocognitive functions. The exclusion criteria were: existing or previous psychiatric disease (bipolar disorder and schizophrenia), neurological disease (alcoholic encephalopathy and epilepsy), cerebrovascular disease, Parkinson disease, or other neurodegenerative disease, the use of any medication, such as antianxiety and sedative drugs, that would affect the mental state during the trial, and a history of allergy to chicken. For understanding the emotional status of the participants at the baseline, the Beck depression inventory (BDI) and Beck anxiety inventory (BAI) were implemented.

### EC and Placebo

BEC and the placebo had a similar appearance and taste. BEC (70 mL), provided by Cerebos Pacific Ltd, Singapore, contains 83 mg of protein and peptide, 3.1 mg of free amino acids, 0.8 mg of hexose, 0.4 mg of fat, and 3 mg of caramel.^25^ It also contains the active dipeptides carnosine (b-alanyl-l-histidine) and anserine (b-alanyl-l-methyl-l-histidine).^15,22^ The placebo (70 mL) contained 83 mg of milk casein and 3 mg of caramel and thus had a protein content, caloric content, and color similar to those of the BEC.

### Neurocognitive Function Assessment

Clinical neuropsychologists blinded to the participant groups performed neurological assessments. The neurocognitive functions of the participants were assessed using a neuropsychological test battery comprising the form-color associative memory test, continuous performance test, word list learning test, digit cancellation test, digit span subtest of the Wechsler Memory Scale (WMS-III), letter number sequencing subtest of the WMS-III, paced auditory serial addition test, and digit vigilance test. Neurocognitive functions such as sustained attention, vigilance, working memory, executive functions (mental flexibility and control, attention division and disinhibition), and short-term memory (registration, delayed recall, and retention) were assessed (Table 1).

**TABLE 1:**
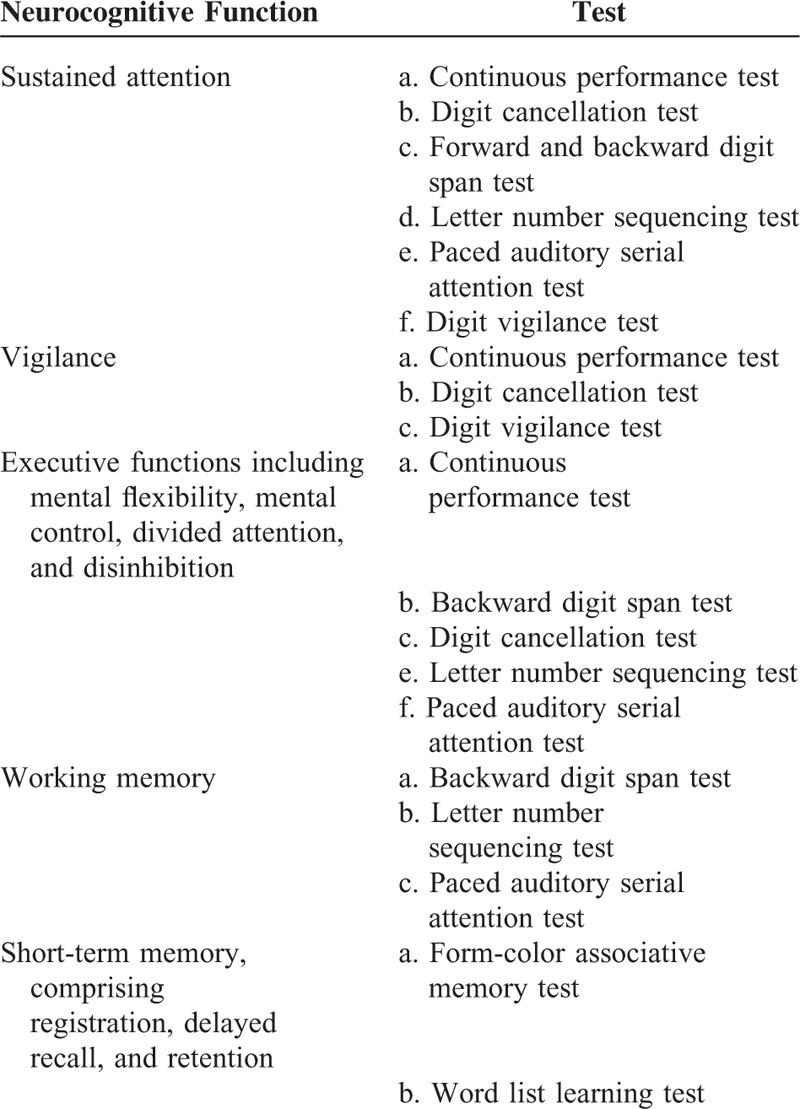
Neurocognitive Functions Surveyed in This Study and the Respective Tests

### Blood Tests

Fasting glucose, renal function (blood urea nitrogen [BUN], creatinine), liver function (glutamate oxaloacetate transaminase, glutamic-pyruvic transaminase [GPT]), ACTH, cortisol, and melatonin levels, and the ratio of classification determinant 4^+^ cells/classification determinant 8^+^ cells (CD4^+/^CD8^+^) were measured in the peripheral blood of the participants at approximately 8:00 am at each visit.

### Statistical Analysis

Demographic characteristics and baseline biochemistry profiles were compared between the BEC and control groups, by using the Student *t* test for continuous variables and the Chi-square test for categorical variables. Generalized estimation equation models were used for comparing neurocognitive function and biochemistry profiles between the groups. To account for multiple comparisons, we calculated the false discovery rate using the Benjamini–Hochberg procedure.^26^ All statistical analyses were performed using SAS Version 9.4 (SAS Institute Inc., Cary, NC) considering 2-sided probabilities.

## RESULTS

The demographic information of the participants is listed in Table 2. All 102 participants, 51 in each group, completed the trial and no adverse events were noted. No differences in gender, education, and ISPJSS, BDI, and BAI scores were observed between the groups. The mean age of the participants in the BEC group (34.1 ± 4.4 years, mean ± standard deviation) was significantly higher than that in the control group (32.2 ± 4.2 years). There are no differences between groups in all the blood tests except melatonin in the baseline. The mean melatonin concentration of BEC group, 16.9 ± 12.9 pg/mL, is higher than that of control group, 12.6 ± 6.1 pg/mL, *P* < 0.05. Table 3 lists the outcomes of neurocognitive function assessments in the 2 groups. No significant changes in the neurocognitive test results were observed in the 2 groups after multiple testing. Table 4 illustrates changes in biochemical values in the BEC and control groups. No differences were observed in the blood tests after treatment compared with those at the baseline or between the 2 groups.

**TABLE 2:**
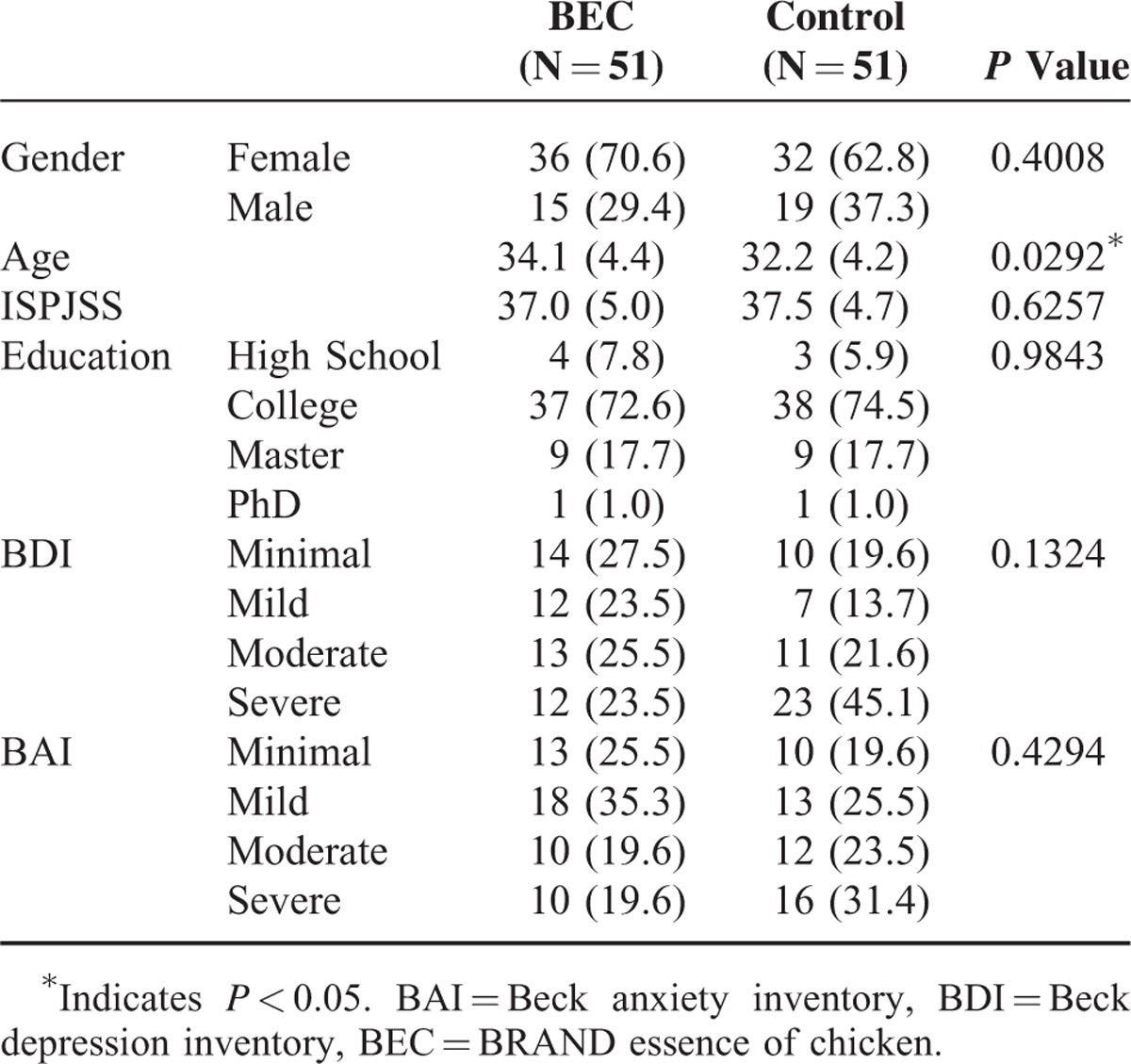
Demographics and Baseline Anxiety, Depression Profiles of the Participants

**TABLE 3:**
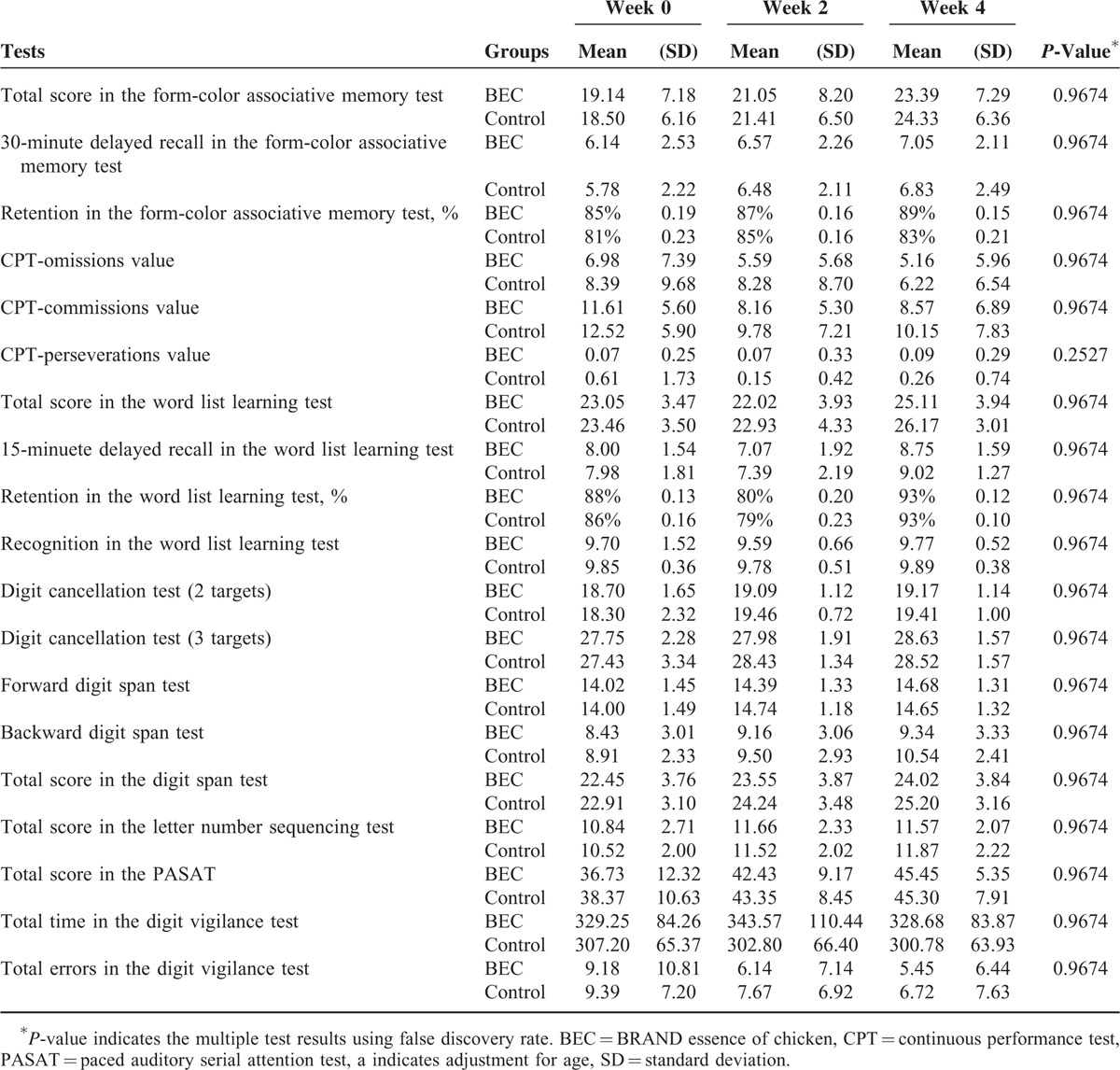
Neurocognitive Function Survey of the Participants

**TABLE 4:**
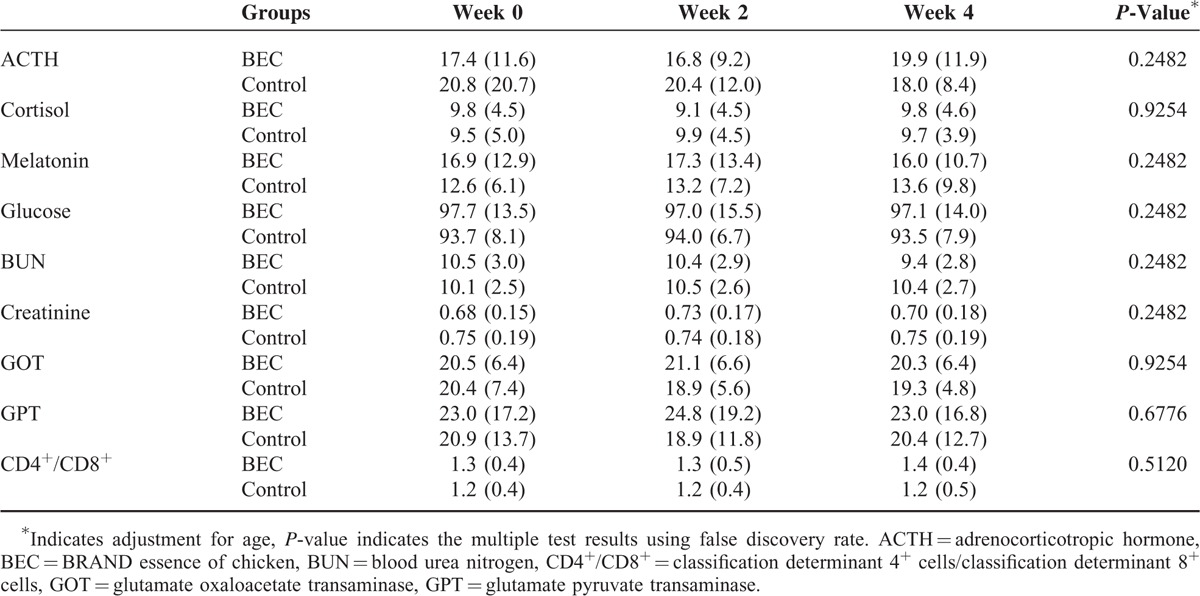
Biochemistry Profiles at Weeks 0, 2, and 4

**FIGURE 2:**
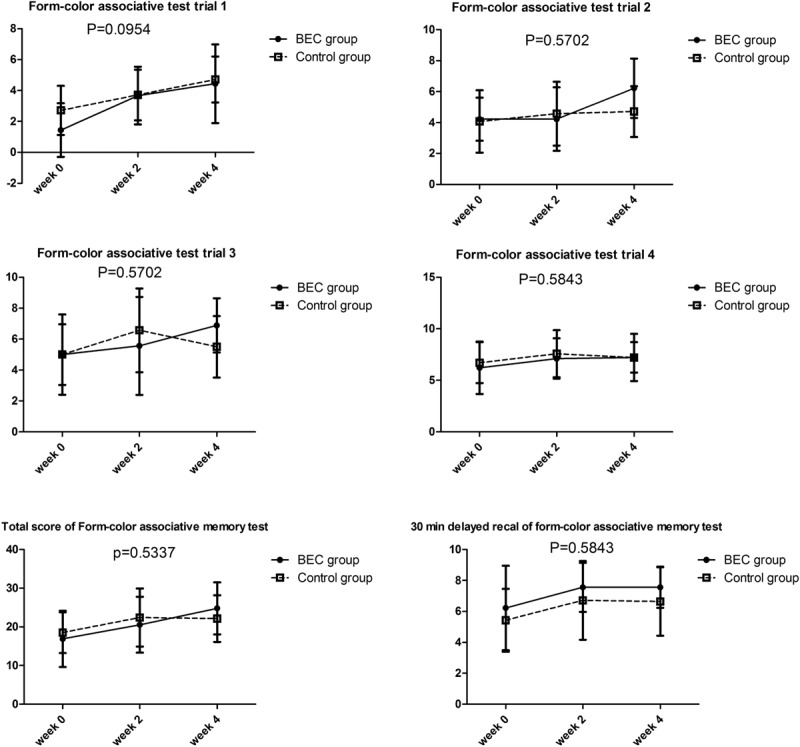
Mean changes in the form-color associative memory test from week 0 to week 4 in participants with high anxiety scores.

To understand the effects of BEC on the neurocognitive functions in participants with high mood disturbance, we analyzed participants with high anxiety scores in the BAI (total score > 25) or high depression scores in the BDI (score > 28). Among the participants with high anxiety scores, 9 received BEC and 14 received the placebo. In the BEC group, the mean age was 34.1 ± 5.0 years and the female/male ratio was 7/2; in the control group, the mean age was 31.7 ± 4.8 years and the female/male ratio was 9/5. The baseline values of all form-color associative memory tests were similar between BEC and control groups. Figure 2 reveals that performance in trial 1 of the form-color associative memory test borderline significantly improved in the BEC group among the participants with high anxiety scores after multiple test correction (*P* = 0.0954).

**FIGURE 3:**
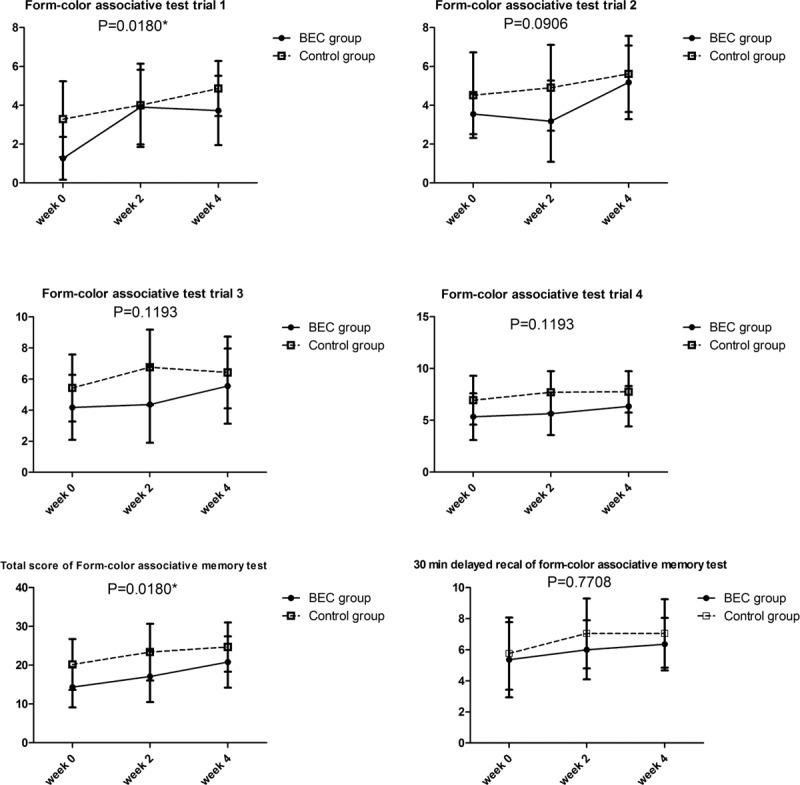
Mean change in the form-color associative memory test from week 0 to week 4 in participants with high depression scores.

Among the participants with high depression scores, 11 received BEC and 21 received the placebo. In the BEC group, the mean age was 35.2 ± 4.9 years and the female/male ratio was 7/4; in the control group, the mean age was 32.0 ± 4.0 years and the female/male was 13/8. The baseline values of all form-color associative tests were about the same between these 2 groups. The BEC group exhibited significantly superior performance in trial 1 (*P* < 0.018), and total score of the form-color associative memory test than the control group after multiple testing (*P* < 0.018) (Figure 3).

## DISCUSSION

WS affects neurocognitive functions, but effective treatments for promoting resilience and resistance to stress-associated effects and maintaining neurocognitive functions are lacking. EC has been widely studied and empirical data suggest that EC is beneficial to multiple organ systems and can improve attention and short-term memory under extremely stressful conditions. In this randomized double-blind trial, we recruited participants with high WS according to ISPJSS scores (>36 for job leaders and 33 for nonleaders). Comprehensive neurocognitive function assessment, blood biochemistry examination, and immunity surveys were implemented at the baseline (week 0), after EC treatment for 2 weeks (week 2), and 2 weeks after the treatment (week 4). To the best of our knowledge, the present study is the 1st randomized double-blind trial for assessing the clinical application of EC.

Multiple neurocognitive function domains, such as attention or vigilance, working memory, immediate and delayed recall of memory, learning effects, retention of memory, and executive functions, were examined in this trial. Almost all neurocognitive functions improved in week 2 and week 4 in both groups. This improvement could be attributed to learning effects attributable to short intervals between the tests (Table 3). However, participants in the BEC group with high depression scores exhibited greater improvement in trials 1 and 2 of the form-color associative test and total scores than those in the control group did. In addition, participants in the BEC group with high anxiety scores exhibited greater improvement in trial 1 of the form-color associative test than those in the control group did. Therefore, we conclude that BEC did not induce significant improvement of neurocognitive functions when we pooled all participants; however, BEC improved the short-term memory of the participants with high depression scores and slightly improved the short-term memory of the participants with high anxiety scores. The form-color memory associative memory test depends on sound short-term memory functions. Stable concentration, working memory, integrated ability, mental control, and an applicable strategy are other factors evaluated in this test. In trial, the participants had to learn extensive and unrelated information immediately and hence may have felt extremely nervous and anxious, which are called the 1st-trial effects. An anxious participant may face difficulty in managing the 1st-trial effects, resulting in monitor errors. Therefore, it is speculated that BEC might be beneficial for controlling inner anxiety under highly stressful conditions in people with high anxiety scores.

In previous studies, BEC improved cognitive function and attention in a short-term mental fatigue scenario.^20,27^ Another study reported that BEC improved cognitive functions such as attention and memory in medical students.^23^ Through near-infrared spectroscopy, BEC was determined to increase oxyhemoglobin concentrations in several regions of the prefrontal areas of the brain, and this area appears to play a fundamental role in short-term memory and working memory. These changes might contribute to the improvement of neurocognitive function.^28^ However, our study was a short-term study, and the effects of long-term BEC use and the mechanisms through which BEC induces neurocognitive improvement remain unknown. Neurocognitive function improved in participants with WS and concomitant depression in this trial. Thus, whether BEC-induced improvement in neurocognitive function increases work efficiency and reduces errors at the workplace in highly stressed workers warrants further investigation.

BEC did not alter the blood biochemistry profile, including liver function; ACTH, cortisol, melatonin, and blood sugar levels; and immunity (examined according to the CD4^+/^CD8^+^ ratio). BEC significantly increased creatinine levels from 0.68 to 0.73 mg/dL, and they returned to 0.70 mg/dL after discontinuation of BEC use. BUN levels decreased in week 4 compared with the baseline data. In an animal study, EC reduced creatinine and BUN levels.^15^ Increased creatinine levels may be caused by various factors. EC contains arginine and glycine, which increase creatine production in the muscle cells, and creatine can be metabolized to creatinine. Blood GPT levels slightly decreased in week 4. The effects of BEC on increasing GPT, creatinine, and BUN levels have not been reported earlier. As GPT, creatinine, and BUN levels were within normal ranges, the clinical significance and long-term effects of BEC on GPT, BUN, and creatinine remain uncertain.

There are a few limitations in this study. First, the intervals between the neurocognitive tests were short. Learning effects may occur and might interfere with the real effects of BEC, although BEC demonstrated its benefits on neurocognitive improvement in this study, particularly for short-term memory. Second, the precise effects of WS on neurocognitive function were not measured. All participants were recruited according to WS scores; however, the influence of WS on neurocognitive functions might be variable. The number of participants in this trial was relatively high; therefore, individual variation might be neglected. Third, the BEC treatment in this trial was short, and the long-term effects of BEC warrant further investigation. Imbalance was observed in the number of participants with high mood disturbance in the BEC and control groups. This study was a double blind randomized trial; therefore, the results were neither unpredictable nor preventable.

In summary, this randomized double-blind trial of young workers under high work stress revealed that BEC treatment for 2 weeks significantly improved short-term memory in the participants with high depression scores and slightly increased GPT and creatinine levels and reduced BUN levels.

## References

[1] Goodnite PM Stress: a concept analysis. *Nurs Forum* 2014; 49:71–74. 24456555 10.1111/nuf.12044

[2] Holmes S Work-related stress: a brief review. *J R Soc Promot Health* 2001; 121:230–235. 11811093 10.1177/146642400112100406

[3] McGrath JE Dunnette MD Stress and behavior in organizations. *Handbook of Industrial and Organizational Psychology*. Chicago: Rand McNally; 1976 1351–1395.

[4] AbuAlRub RF Job stress, job performance, and social support among hospital nurses. *J Nurs Scholarsh* 2004; 36:73–78. 15098422 10.1111/j.1547-5069.2004.04016.x

[5] Gandi JC Wai PS Karick H The role of stress and level of burnout in job performance among nurses. *Ment Health Fam Med* 2011; 8:181–194. 22942900 PMC3314275

[6] Lue BH Chen HJ Wang CW Stress, personal characteristics and burnout among first postgraduate year residents: a nationwide study in Taiwan. *Med Teach* 2010; 32:400–407. 20423259 10.3109/01421590903437188

[7] Sandström A Peterson J Sandström E Cognitive deficits in relation to personality type and hypothalamic-pituitary-adrenal (HPA) axis dysfunction in women with stress-related exhaustion. *Scand J Psychol* 2011; 52:71–82. 20964695 10.1111/j.1467-9450.2010.00844.x

[8] Blix E Perski A Berglund H Long-term occupational stress is associated with regional reductions in brain tissue volumes. *PLoS One* 2013; 8:e64065. 23776438 10.1371/journal.pone.0064065 PMC3679112

[9] Mommersteeg PM Heijnen CJ Kavelaars A Immune and endocrine function in burnout syndrome. *Psychosom Med* 2006; 68:879–886. 17079708 10.1097/01.psy.0000239247.47581.0c

[10] von Känel R Bellingrath S Kudielka BM Association between burnout and circulating levels of pro- and anti-inflammatory cytokines in schoolteachers. *J Psychosom Res* 2008; 65:51–59. 18582612 10.1016/j.jpsychores.2008.02.007

[11] Yang Y Koh D Ng V Self perceived work related stress and the relation with salivary IgA and lysozyme among emergency department nurses. *Occup Environ Med* 2002; 59:836–841. 12468751 10.1136/oem.59.12.836 PMC1763606

[12] Li YF He RR Tsoi B Bioactivities of chicken essence. *J Food Sci* 2012; 77:R105–R110. 22432477 10.1111/j.1750-3841.2012.02625.x

[13] Huang WC Lin CI Chiu CC Chicken essence improves exercise performance and ameliorates physical fatigue. *Nutrients* 2014; 6:2681–2696. 25045938 10.3390/nu6072681 PMC4113764

[14] Man YC Yee CW Shing WK The enhancing effects of a chicken-meat extract on serum Ig concentrations in normal and scalded animals. *Br J Nutr* 2005; 94:51–55. 16115332 10.1079/bjn20051449

[15] Matsumura Y Kita S Ono H Preventive effect of a chicken extract on the development of hypertension in stroke-prone spontaneously hypertensive rats. *Biosci Biotechnol Biochem* 2002; 66:1108–1110. 12092823 10.1271/bbb.66.1108

[16] Geissler C Boroumand-Naini M Harada M Chicken extract stimulates haemoglobin restoration in iron deficient rats. *Int J Food Sci Nutr* 1996; 47:351–360. 8844257 10.3109/09637489609041035

[17] Wu T Watanabe H Hong LK Effect of BRAND's essence of chicken on the resetting process of circadian clocks in rats subjected to experimental jet lag. *Mol Biol Rep* 2011; 38:1533–1540. 20835889 10.1007/s11033-010-0261-5

[18] Xu M He RR Zhai YJ Effects of carnosine on cyclophosphamide-induced hematopoietic suppression in mice. *Am J Chin Med* 2014; 42:131–142. 24467540 10.1142/S0192415X14500098

[19] Xu CL Sim MK Effect of oral feeding of essence of chicken on the level of 5-hydroxyindole acetic acid in the cerebrospinal fluid of the rat. *Int J Food Sci Nutr* 1997; 48:113–117. 9135774 10.3109/09637489709006970

[20] Nagai H Harada M Nakagawa M Effects of chicken extract on the recovery from fatigue caused by mental workload. *Appl Human Sci* 1996; 15:281–286. 10.2114/jpa.15.281 9008982

[21] Lü YQ He RR Watanabe H Effects of a chicken extract on food-deprived activity stress in rats. *Biosci Biotechnol Biochem* 2010; 74:1276–1278. 20530894 10.1271/bbb.90950

[22] Kurihara H Yao X Nagai H Anti-stress effect of BRAND's essence of chicken (BEC) on plasma glucose levels in mice loaded with restraint stress. *J Heal Sci* 2006; 52:252–258.

[23] Zain AM Syedsahiljamalulail S Effect of taking chicken essence on stress and cognition of human volunteers. *Malays J Nutr* 2003; 9:19–29. 22692529

[24] Institute of Occupational Safety and Health, Council of Labor Affairs, Executive Yuan, R. O. C. (Taiwan). The Technical Handbook for the Evaluation of Labor Occupational Stress. 1999, IOSH88-T-028.

[25] Chao JC Tseng HP Chang CW Chicken extract affects colostrum protein compositions in lactating women. *J Nutr Biochem* 2004; 15:37–44. 14711459 10.1016/j.jnutbio.2003.09.009

[26] Benjamini Y Hochberg Y Controlling the false discovery rate: a practical and powerful approach to multiple testing. *JR Stat Soc Ser B* 1995; 57:289–300.

[27] Yamano E Tanaka M Ishii A Effects of chicken essence on recovery from mental fatigue in healthy males. *Med Sci Monit* 2013; 19:540–547. 23831862 10.12659/MSM.883971 PMC3707410

[28] Konagai C Watanabe H Abe K Effects of essence of chicken on cognitive brain function: a near-infrared spectroscopy study. *Biosci Biotechnol Biochem* 2013; 77:178–181. 23291775 10.1271/bbb.120706

